# Methamphetamine induces alterations in the long non-coding RNAs expression profile in the nucleus accumbens of the mouse

**DOI:** 10.1186/s12868-015-0157-3

**Published:** 2015-03-25

**Authors:** Li Zhu, Jie Zhu, Yufeng Liu, Yanjiong Chen, Yanlin Li, Liren Huang, Sisi Chen, Tao Li, Yonghui Dang, Teng Chen

**Affiliations:** College of Forensic Medicine, Xi’an Jiaotong University Health Science Center, Xi’an, Shaanxi 710061 PR China; The Key Laboratory of Health Ministry for Forensic Science, Xi’an Jiaotong University, Shaanxi, PR China; Beijing Genomics Institute, Shenzhen, 518083 PR China; Departments of Immunology and Pathogenic Biology, Xi’an Jiaotong University Health Science Center, Xi’an, Shaanxi 710061 PR China

**Keywords:** Methamphetamine, Sensitization, Nucleus accumbens, LncRNA, *Cis*, *Trans*

## Abstract

**Background:**

Repeated exposure to addictive drugs elicits long-lasting cellular and molecular changes. It has been reported that the aberrant expression of long non-coding RNAs (lncRNAs) is involved in cocaine and heroin addiction, yet the expression profile of lncRNAs and their potential effects on methamphetamine (METH)-induced locomotor sensitization are largely unknown.

**Results:**

Using high-throughput strand-specific complementary DNA sequencing technology (ssRNA-seq), here we examined the alterations in the lncRNAs expression profile in the nucleus accumbens (NAc) of METH-sensitized mice. We found that the expression levels of 6246 known lncRNAs (6215 down-regulated, 31 up-regulated) and 8442 novel lncRNA candidates (8408 down-regulated, 34 up-regulated) were significantly altered in the METH-sensitized mice. Based on characterizations of the genomic contexts of the lncRNAs, we further showed that there were 5139 differentially expressed lncRNAs acted via *cis* mechanisms, including sense intronic (4295 down-regulated and one up-regulated), overlapping (25 down-regulated and one up-regulated), natural antisense transcripts (NATs, 148 down-regulated and eight up-regulated), long intergenic non-coding RNAs (lincRNAs, 582 down-regulated and five up-regulated), and bidirectional (72 down-regulated and two up-regulated). Moreover, using the program RNAplex, we identified 3994 differentially expressed lncRNAs acted via *trans* mechanisms. Gene ontology (GO) and KEGG pathway enrichment analyses revealed that the predicted *cis-* and *trans-* associated genes were significantly enriched during neuronal development, neuronal plasticity, learning and memory, and reward and addiction.

**Conclusions:**

Taken together, our results suggest that METH can elicit global changes in lncRNA expressions in the NAc of sensitized mice that might be involved in METH-induced locomotor sensitization and addiction.

**Electronic supplementary material:**

The online version of this article (doi:10.1186/s12868-015-0157-3) contains supplementary material, which is available to authorized users.

## Background

It has been reported that repeated exposure to drugs of abuse results in long-lasting behavioural changes such as locomotor sensitization that are thought to be due to structural and functional changes in associated brain regions, particularly the NAc [1,2]. Previous studies have indicated that addictive drugs induce persistent and dynamic cellular and molecular modifications accompanied by distinct processes of drug addiction [3-6]. The complexity of drug-induced stable changes suggests that synchronized programs of gene regulation might be executed during drug addiction [7]; however, the precise mechanism remains unclear.

Previous research has shown that addictive drugs induce aberrant expression of non-coding RNAs (ncRNAs) [8,9], the function of which are thought to be among the most important mechanisms underlying gene regulation [10,11]. For example, increasing evidence has demonstrated that microRNAs play an important role in modulating the potency of addictive drugs by mediating the expressions of target genes [7,12,13]. However, the potential effects of lncRNA on drug addiction remain largely unknown.

LncRNAs are defined as transcripts longer than 200 nt that lack the ability to encode protein products [14]. As transcriptional modulators and epigenetic regulators, lncRNAs have been found to regulate the expressions of proximal and distal protein-coding genes through *cis*- and *trans*-acting mechanisms [11]. Emerging evidence has implicated lncRNAs in neuroplasticity, brain development, neurodegenerative, and neuropsychiatric disorders [15-18], together, this evidence suggests a meaningful role of lncRNAs in brain diseases including drug addiction. Using microarrays, recent studies have revealed that cocaine and heroin induce widespread alterations of lncRNAs in the NAc of cocaine-conditioned mice and heroin addicts [9,19],which suggests that lncRNAs might play an important role in the regulation of drug addiction. However, all of these studies targeted small numbers of candidate lncRNAs and therefore could not identify unknown lncRNAs and did not provide a complete spectrum of drug-induced changes in lncRNA levels.

To investigate the expression profiles of lncRNAs and their potential effects on METH-induced locomotor sensitization in the current study, we examined the alterations in lncRNAs expression profiles in the NAc of METH-sensitized mice via the transcriptomics-based approach, ssRNA-seq. We found that METH elicited global changes in lncRNAs expression in the NAc of mice and that predicted *cis-* and *trans-*associated genes were significantly enriched during neuronal development, neuronal plasticity, learning and memory, and reward and addiction. Our results suggest that lncRNAs might be involved in the regulation of expression of associated genes and thus contribute to METH-induced locomotor sensitization and addiction.

## Results

### SsRNA-seq summary

Complementary DNA samples generated from RNA that was extracted from the NAc lysates of saline and METH-treated mice were measured with ssRNA-seq. A total of 49.62 million and 50.33 million clean reads were obtained from the saline and METH groups of mice, respectively. The clean reads of each group were then separately aligned to the mouse genome (UCSC mm9) [20] and 84.02% (saline) and 80.82% (METH) reads were mapped to the reference genome, which included 28.65 million (saline) and 27.08 million (METH) perfectly matched reads (Table 1). Additionally, among the total mapped reads, there were 36.76 million (74.09%) and 33.14 million (65.84%) uniquely matched reads that were obtained from the saline and METH groups of mice, respectively (Table 1). All of the mapped reads were then assembled and annotated. Consequently, 25677 and 23579 lncRNAs were obtained from the saline and METH groups of mice, respectively, by alignment to the database of non-coding RNAs (NONCODE v3.0) [21] (Table 2). The analysis of the relative expression levels (RPKM) of these known lncRNAs revealed that the vast majority of the lncRNAs (71.31% and 78.35% of the total known lncRNA transcripts in saline and METH groups of mice respectively) were expressed at the level of RPKM < 5 (Table 2). Moreover, we also identified 17860 and 15965 novel lncRNA candidates in the saline and METH groups, that could not matched to any sequences that correspond to known lncRNAs or protein-coding transcripts.

**Table 1:** **Statistical alignment of the sequencing data from the saline and METH groups**

	**Saline**	**METH**
Total clean reads	49621072 (100%)	50330816 (100%)
Total mapped reads	41689728 (84.02%)	40675732 (80.82%)
Perfect match^a^	28650476 (57.74%)	27082591 (53.81%)
≤5 bp mismatch^b^	13039252 (26.28%)	13593141 (27.01%)
Unique match^c^	36764592 (74.09%)	33138073 (65.84%)
Multi-position match^d^	4925136 (9.93%)	7537659 (14.98%)
Total unmapped reads^e^	7931344 (15.98%)	9655084 (19.18%)

^a^indicates clean reads that aligned without mismatch in the total mapped reads.

^b^indicates clean reads that aligned with fewer than 5 bp mismatches in the total mapped reads.

^c^represents the mapped reads that aligned to only one position in the mouse genome.

^d^represents the mapped reads that aligned to more than one positions in the mouse genome.

^e^indicates reads that did not match to the mouse genome.

**Table 2:** **Distribution of known lncRNAs detected by ssRNA-seq**

	**Saline**	**METH**
Known lncRNAs^a^	25677 (100%)	23579 (100%)
<5 RPKM^b^	18309 (71.31%)	18475 (78.35%)
5 ~ 100 RPKM^c^	6899 (26.87%)	4638 (19.67%)
≥100 RPKM^d^	469 (1.83%)	466 (1.98%)

^a^represents lncRNA transcripts that were matched with NONCODE v3.0

^b^indicates the known lncRNAs that were expressed at a level below 5 RPKM.

^c^indicates the known lncRNAs that were expressed at a level between 5 and 100 RPKM.

^d^indicates the known lncRNAs that were expressed at a level greater than 100 RPKM.

### METH- induced aberrant expressions of lncRNAs in the NAc of mice

**Figure 1:**
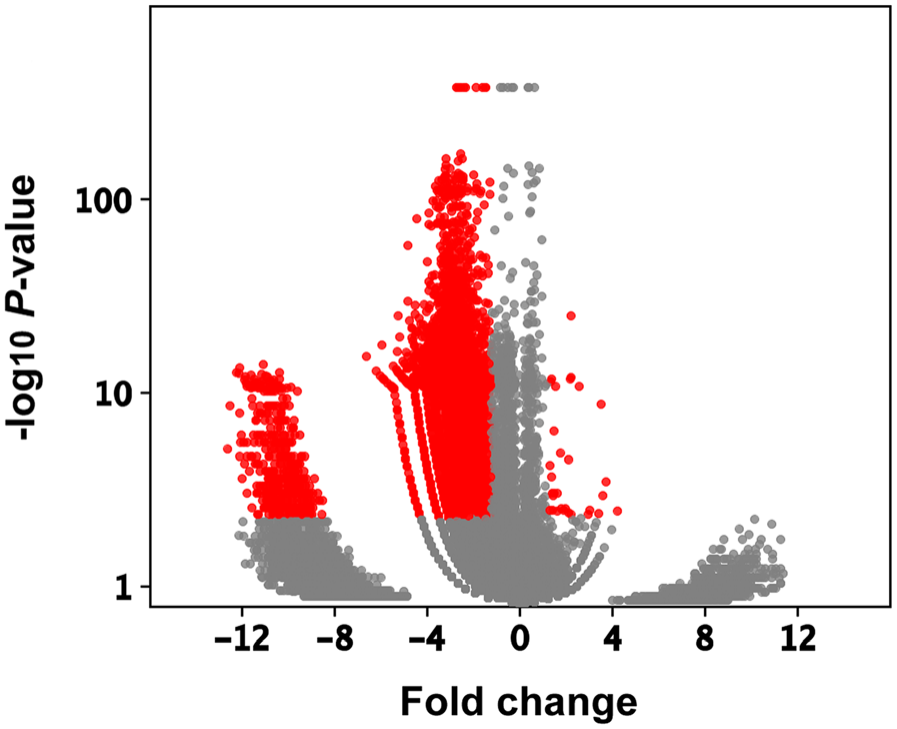
**METH-induced global changes in lncRNA expressions in the NAc of sensitized mice.** Volcano plots providing *P* values and fold change of known lncRNAs. Red point: the differentially expressed lncRNAs at the level of *P* < 0.001, FDR ≤ 0.0001 and an absolute value of the fold change ≥ 1.25.

To further identify the differentially expressed lncRNAs in the NAc of the METH-sensitized mice, the RPKM ratios of the lncRNAs in each group were subjected to a log-2 transform to produce fold changes and threshold based on a combination of statistical significance (*P* < 0.001, FDR ≤ 0.0001) and the absolute value of the fold change (≥1.25) was set. We found that 6246 known lncRNAs exhibited significantly altered expressions in the NAc of the METH-sensitized mice that included 31 up-regulations and 6215 down-regulations (Additional file 1). A volcano plot illustrated the variance in the lncRNAs numbers at different *P*-values and fold changes (Figure 1).

Additionally, analysis of the novel lncRNA candidates revealed that 8442 novel lncRNA candidates exhibited significantly different expressions (*P* < 0.001, FDR ≤ 0.0001 and absolute value of the fold change ≥ 1.25) that included 34 increases and 8408 decreases (data were not shown).

### Validation of lncRNAs expression by quantitative real-time PCR

**Figure 2:**
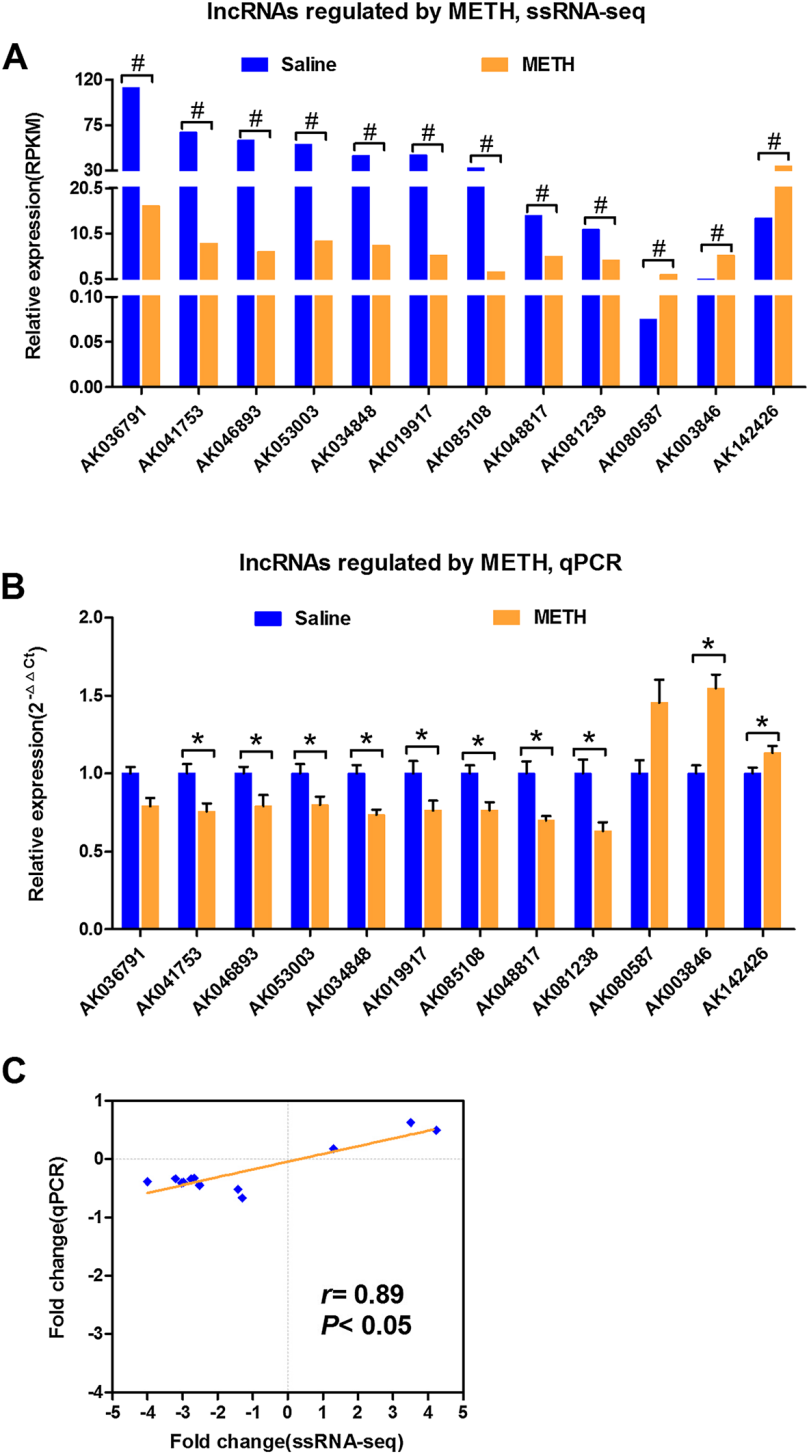
**QPCR confirmations of the differential expressions of selected lncRNAs in METH-sensitized mice. A**, Differentially expressed lncRNAs as detected by ssRNA-seq. 12 significantly altered lncRNAs are shown with the corresponding RPKM. # indicates the following significance cutoff: *P* < 0.001, FDR ≤ 0.0001 and an absolute fold change value ≥1.25. **B**, Validation of differentially expressed lncRNAs by qPCR. The expressions of the 12 selected lncRNAs were detected by qPCR. The expression levels were calculated relative to GAPDH. The values are presented as the means ± the SEMs. The differences between the saline and METH groups were statistically tested with independent-sample *t*-tests. *P < 0.05 compared to saline, n = 11-15. **C**, The ssRNA-seq and qPCR correlations are shown above. Pairwise scatterplots comparing the fold changes (log_2_ METH/saline) of the selected lncRNAs in the NAc as computed from the ssRNA-seq data (horizontal axis) and the qPCR data (vertical axis). The Pearson’s Coefficient is represented as the linear correlation coefficient, *r*.

To validate the METH-induced changes in lncRNA expression that were detected by ssRNA-seq, 12 differentially expressed lncRNAs were randomly selected, and their expressions were then examined with quantitative real-time PCR (qPCR). As shown in Figure 2, 10 of the selected lncRNAs were significantly changed (eight down-regulated and two up-regulated) in METH-sensitized mice as detected by both ssRNA-seq (Figure 2A, #*P* < 0.001, FDR ≤ 0.0001 and an absolute value of the fold change ≥ 1.25 compared to the saline group of mice) and qPCR (Figure 2B, *P < 0.05 compared to the saline group of mice, n = 11-15 per group). Although the expression of AK036791 and AK080587 detected by qPCR were not significantly regulated, they showed similar expression trends in qPCR and ssRNA-seq. Moreover, a strong agreement across the two methods was observed in that the results of the qPCR were similar to those obtained from the ssRNA-seq analyses (Figure 2C, *r* = 0.89, *P* < 0.05). These data indicated the good reproducibility of the observed expression changes in the lncRNAs based on an independent method.

### Genomic characterization of differentially expressed known lncRNAs

**Figure 3:**
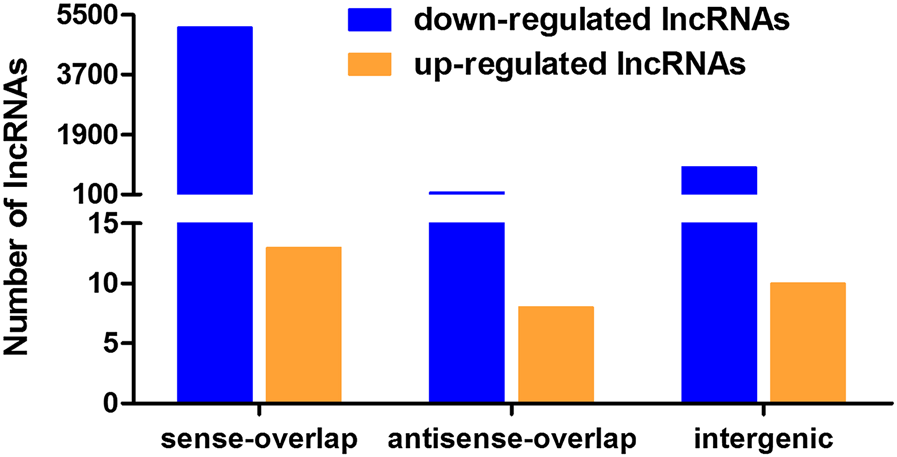
**Genomic characterizations of the differentially expressed lncRNAs.** Sense-overlap: lncRNAs that overlapped with the protein-coding genes that were transcribed from the same strand. Antisense-overlap: lncRNAs that overlapped with the protein-coding genes that were transcribed in the opposite direction. Intergenic lncRNAs: lncRNAs with transcriptional units that were separate from protein-coding genes. The numbers of each type of lncRNAs are shown (y-axis).

To predict the potential role of lncRNAs in the regulation of the expressions of protein-coding genes, we next investigated the genomic context of the differentially expressed known lncRNAs. LncRNAs can be classified into intergenic lncRNAs, sense-overlap lncRNAs and antisense-overlap lncRNAs based on their locations relative to protein-coding genes. Here, we identified 5141 sense-overlap (5128 down-regulated and 13 up-regulated), 172 antisense-overlap (164 down-regulated and eight up-regulated) and 933 intergenic (923 down-regulated and 10 up-regulated) lncRNAs among the differentially expressed known lncRNAs that we detected (Figure 3).

### LncRNAs in *cis* regulation

**Figure 4:**
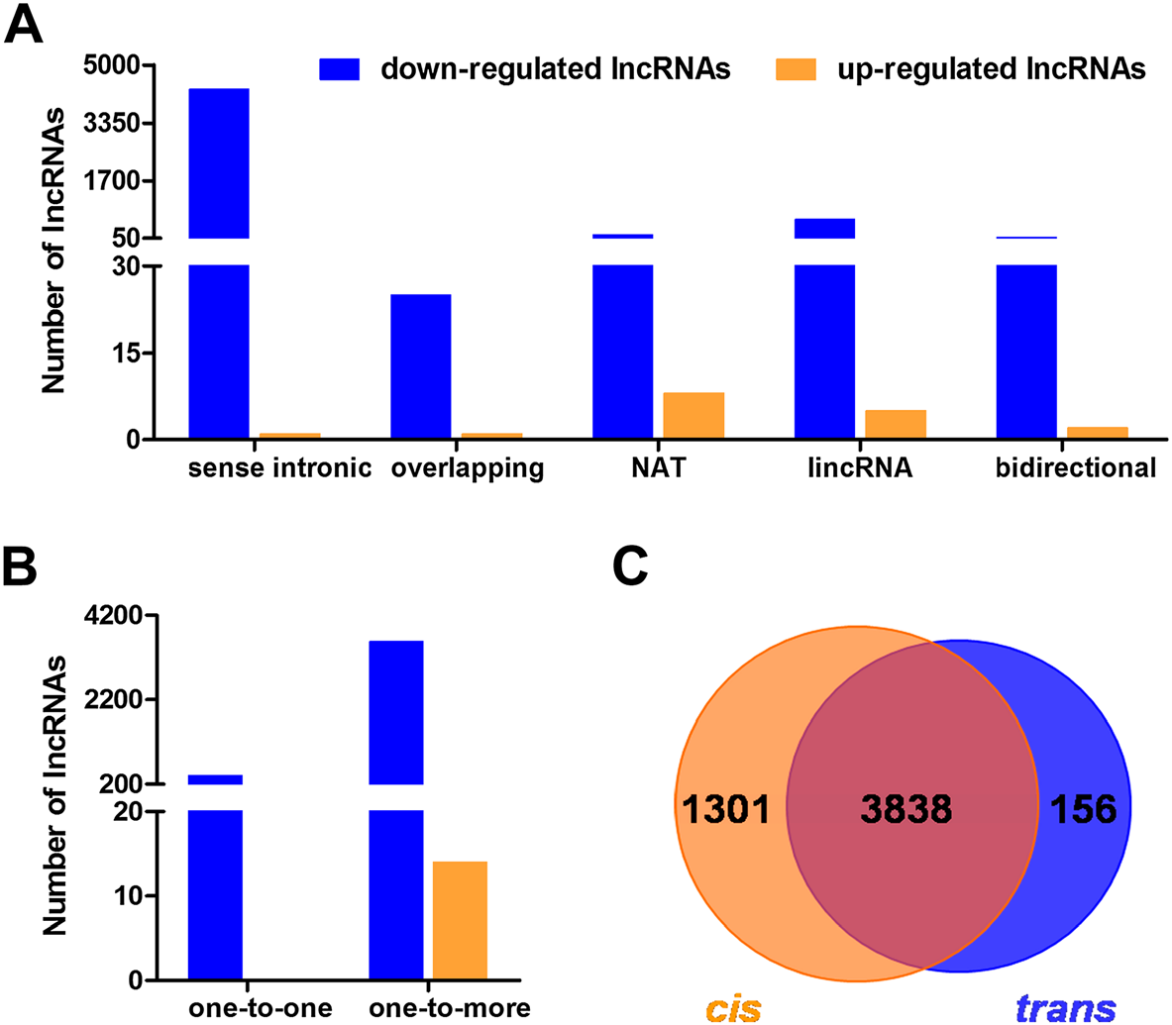
***Cis*** **and** ***trans*** **analyses of the differentially expressed lncRNAs. A**, *Cis* analyses of the differentially expressed known lncRNAs. *Cis*-acting lncRNAs are classified into sense intronic, overlapping, NAT, lincRNA and bidirectional (x-axis) and the numbers of each type of *cis*-acting lncRNAs are shown (y-axis). **B**, *Trans* analyses of the differentially expressed known lncRNAs. The differentially expressed lncRNAs could exhibit *trans* interactions with associated genes in one-to-one (a single lncRNA had one associated gene) or one-to-more (a single lncRNA had more than one associated genes) manners (x-axis). The numbers of each type of *trans*-acting lncRNAs are shown (y-axis). **C**, Overlap of the *cis*- and *trans*-acting lncRNAs.

LncRNAs can regulate the expressions of genes that are located on that same chromosome, and such regulation is called *cis* regulation [11]. Based on their genomic localization relative to nearby protein-coding genes, lncRNAs can be further classified as sense intronic, overlapping, NAT, lincRNA and bidirectional, and these classes of lncRNAs have been reported to regulate their protein-coding host genes in *cis* manners [22,23]. In the present study, we subjected the differentially expressed known lncRNAs to *cis* analysis, and we found that 82% (5139 of 6246) of the differentially expressed known lncRNAs could act in a *cis* manner, including 4296 sense intronic lncRNAs, 26 overlapping lncRNAs, 156 NATs, 587 lincRNAs and 74 bidirectional lncRNAs (Figure 4A).

### Sense intronic lncRNAs

Sense intronic lncRNAs originate from long introns that are transcribed from the same strand as the associated protein-coding genes. The sense intronic lncRNAs are biologically significant because they have been found to be both co-expressed with their host protein-coding gene and independently expressed, particularly in the mouse brain [24]. In the present study, we identified 4296 known lncRNAs that were located in the introns of protein-coding genes that include 4295 down-regulated and one up-regulated lncRNAs (Figure 4A, Additional file 2).

### Overlapping lncRNAs

Overlapping lncRNAs are lncRNAs contain a protein-coding gene and are transcribed in the same direction as that gene. This type of lncRNA can regulate downstream transcription by opening the chromatin structure, depositing histone marks [25], and *cis*-acting promoter competition [26]. Here, we identified 26 significantly changed lncRNAs that overlapped with protein-coding genes, including 25 decreased and one increased lncRNAs (Figure 4A, Additional file 3).

### NATs

NATs are lncRNAs that are transcribed from the antisense strand of a gene locus, and are overlapping with the RNA that transcribed from the sense strand. LncRNAs of this type have been discovered to be widespread in the mammalian genome and work through multiple mechanisms to regulate the expressions of their sense partners [27,28]. In the present study, we identified 156 differentially expressed NATs that included 148 that were down-regulated and eight that were up-regulated (Figure 4A, Additional file 4). For example, the potassium voltage-gated channel, subfamily Q, member 1, opposite strand/antisense transcript 1 (*Kcnq1ot1*) [Genebank: NR_001461], and the zinc finger homeobox 2, antisense (*Zfhx2as*) [Genebank: AK032589] were found to be significantly down-expressed in the METH-treated mice and have previously been found to modulate the expression of their sense partners [29,30].

### LincRNAs

LincRNAs are found more than 10 kb away from any nearby protein-coding locus [31,32]. A possible working model of the role of lincRNAs in gene regulation involves their actions as enhancers that activate transcriptional promotion and chromatin looping [33,34]. We identified 587 lincRNAs that were significantly altered in the METH-sensitized mice, including 582 that were down-regulated and five that were up-regulated (Figure 4A, Additional file 5). Several lincRNAs, including nuclear-enriched abundant transcript 2 (*Neat2*) [Genebank: AY722410], nuclear enriched abundant transcript 1 (*Neat1*) [Genebank: GQ859163], and myocardial infarction associated transcript (*Miat*) [Genebank: NR_033657], that were significantly down-regulated by METH have previously been characterized as possessing neurological functions [15,16].

### Bidirectional lncRNAs

Bidirectional lncRNAs are oriented head-to-head with a protein-coding gene within 1 kb, but are transcribed in the opposite direction. Bidirectional lncRNAs have been shown to affect the *cis* regulation of the nearby protein-coding genes potentially via promoter competition or the maintenance of an open chromatin structure [35,36]. We identified 74 aberrantly altered lncRNAs that formed bidirectional pairs with protein-coding genes in the current study (Figure 4A, Additional file 6) that included 72 down-regulated and two up-regulated lncRNAs.

### LncRNAs in *trans* regulation

LncRNAs can work in a *trans* manner when they affect genes on other chromosomes [11]. Previous studies have shown that lncRNAs can interact with associated mRNAs via the formation of complementary hybrids [37,38]. Therefore, using the RNAplex program [39], we subjected the differentially expressed known lncRNAs to *trans*-analysis and found that 64% (3994 of 6246) of the differentially expressed known lncRNAs were capable of acting in a *trans* manner, and 2386 of the associated genes have been found. We further investigated the networks formed by the *trans*-acting lncRNAs and their associated genes, which are termed the ‘many-to-many’ type; i.e., one lncRNA can have one or more associated genes. As shown in Figure 4B, 403 down-regulated lncRNAs exhibited one-to-one *trans*-regulation relationships with protein-coding genes (Additional file 7). In contrast, over 90% lncRNAs (3591 of 3994) might have more than one *trans*-associated gene. Additionally, the *cis*- and *trans*-acting lncRNAs also overlapped, and 3838 known lncRNAs were identified as having both *cis*- and *trans*-associated genes (Figure 4C).

### Functional analyses of the *cis*- and *trans*-associated genes

**Figure 5:**
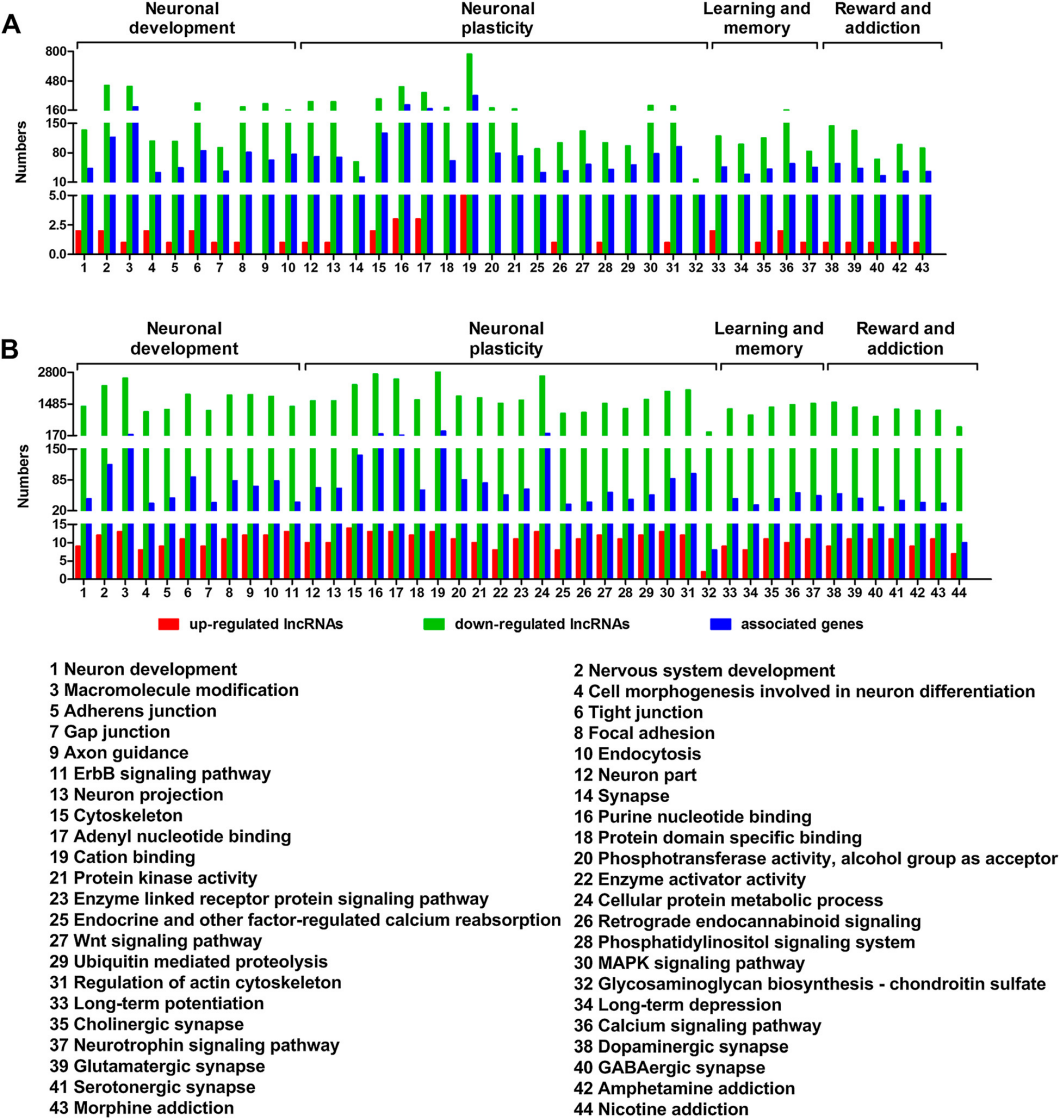
**GO and pathway analyses of the** ***cis*** **- and** ***trans*** **-associated genes. A**, GO and pathway analyses of the *cis*-associated genes; **B**, GO and pathway analyses of the *trans*-associated genes. The significantly enriched GO terms and pathways (P < 0.05 and FDR < 0.05) within certain neurological functions ( 1–44) associated with neuronal development (1–11), neuronal plasticity (12–32), learning and memory (33-37), and reward and addiction ( 38–44), are shown with the numbers of down-regulated lncRNAs, up-regulated lncRNAs, and the associated genes that were identified by *cis* and *trans* analyses, respectively.

To further investigate the potential effects of known lncRNAs on METH-induced locomotor sensitization, we subjected the *cis*- and *trans*-associated genes to GO and KEGG pathway analyses. Using *P* < 0.05 and FDR < 0.05 as cutoff for significance, we found that the predicted *cis* and *trans*-associated genes were significantly enriched in axon guidance, ubiquitin mediated proteolysis, neuron projection, the MAPK signalling pathway, long-term potentiation (LTP), long-term depression (LTD), calcium signalling pathway, dopaminergic synapse, and glutamatergic synapse; these processes are generally linked to neuronal development, neuronal plasticity, learning and memory, and reward and addiction (Figure 5, see full list in Additional files 8 and 9).

## Discussion

Previous studies have found that the expressions of lncRNAs are aberrantly altered in the NAc of cocaine-conditioned mice and heroin addicts, which suggests an important role of lncRNAs in drug addiction [9,19]. Nevertheless, the expression profiles of lncRNAs and their potential effects on METH-induced locomotor sensitization are largely unknown. Here, we used high-throughput ssRNA-seq technology to examine the alteration in the lncRNAs expression profile in the NAc of METH-sensitized mice. Using a stringent threshold for statistical significance (*P* < 0.001, FDR ≤ 0.0001 and an absolute value of the fold change ≥ 1.25), we identified numerous (6246) METH-regulated lncRNAs (Additional file 1), and 125 of these lncRNAs were also significantly altered in the NAc of cocaine-conditioned mice [9]. Interestingly, the differentially expressed lncRNAs were less likely to be up-regulated and more likely to be down-regulated in the METH-sensitized mice. These data are consistent with the pattern of lncRNA expression in the NAc of cocaine-treated mice [9]. Although the precise regulatory mechanism remains unclear, these results suggest that METH might reduce the expressions of lncRNAs. Further studies are needed to investigate the potential role of these differentially expressed lncRNAs. Nevertheless, our findings suggest that lncRNAs might be involved in METH addiction and provide new insight into the molecular mechanisms of METH abuse. To our knowledge, this is the first description of global lncRNAs expression profiling in the context of METH-induced behavioural sensitization. Moreover, our results provide numerous of METH-responsive lncRNA candidates for further functional research.

It has been shown that lncRNAs originate from complex loci that contain interlaced networks of long non-coding and protein-coding transcripts [40,41]. Analysis of the genomic characterization of lncRNAs is helpful in predicting their regulatory effects at the biological level. Indeed, a number of previously characterized lncRNAs have been proven to regulate the expressions of protein-coding genes that share genomic loci with the lncRNAs [30,42,43]. In the present study, we found that lncRNAs were associated with protein-coding genes in a variety of manners that included sense intronic, overlapping, lincRNA, NAT and bidirectional. From the perspective of the lncRNAs in their genomic context, our findings suggest the potential functions of these lncRNAs in terms of METH sensitization. Furthermore, lncRNAs have been reported to be involved in regulation of gene expression through *trans-*acting pathways in which they affect genes on other chromosomes. Here, we showed that numerous of METH-regulated lncRNAs interacted with associated protein-coding genes in *trans* manners, which suggests that these lncRNAs might be biologically meaningful. Notably, the *trans*-acting lncRNAs were found to form a “many-to-many” network with their associated genes, which reflects the complexity of the mechanisms of the regulation of METH-regulated lncRNAs. Interestingly, five lncRNAs (*Kcnq1ot1*, *Zfhx2as*, *Neat1*, *Neat2*, and *Miat*) that were found to be regulated by METH in our study have been reported to interact in *cis* or *trans* manners with targeted loci. For example, the antisense transcripts *Kcnq1ot1* and *Zfhx2as* were found to regulate the expressions of their sense partners [29,30], which have been reported to be involved in the modulation of LTP in the hippocampus [44] and behavioural abnormalities [45], respectively. It has been reported that *Neat1, Neat2* and *Miat* function as cofactors for pre-mRNA splicing by interacting with the splicing factors [46-48], and significantly, *Neat2* appears to regulate neuronal plasticity by modulating the expressions of multiple synaptic genes [15], which suggests that lncRNAs-related nuclear modification might play a role in METH addiction through rapid post-transcriptional changes in gene expression. Notably, a preliminary examination of a published dataset based on heroin abusers revealed up-regulations of *NEAT1*, *NEAT2* and *MIAT* [19], which is suggestive of differential responses across different drugs of abuse. Although the precise regulatory mechanism remains unclear, these well-characterized lncRNAs might play roles in terms of METH abuse. Taken together, the identification of *cis* and *trans*-acting lncRNAs suggests the potential functional implications of METH-regulated lncRNAs that might control the expressions of proximal or distal associated genes and thus contribute to METH-induced locomotor sensitization and addiction.

A major function of lncRNAs appears to be the control of the gene expression via *cis-* and *trans-*acting pathways [11]. Thus, functional analyses of *cis*- and *trans*-associated genes are helpful for predicting the potential effects of lncRNAs on METH-induced locomotor sensitization. In the present study, we found that the predicted *cis*- and *trans*-associated genes were significantly enriched during neuronal development, neuronal plasticity, learning and memory, and reward and addiction (Figure 5). Previous studies have demonstrated that the neuronal plasticity that occurrs as a consequence of exposure to drugs of abuse plays a critical role in the modulation of persistent addictive behaviours [49-51]. Similarly, it has been reported that chronic exposure to drugs of abuse modulates learning and memory, which are thought to underlie rewarding and addictive behaviours [52-54]. Therefore, our findings suggest that the lncRNAs that were modified by METH in this study might influence the expressions of genes that are involved in neuronal plasticity, learning and memory and thus contribute to METH addiction.

Moreover, we unexpectedly found that sense intronic lncRNAs comprised the largest portion of the *cis*-lncRNAs. Previous studies have demonstrated that intronic lncRNAs can either be transcriptional segments of processed mRNAs or independent transcripts that are simply located within intron-annotated genomic regions, and intronic lncRNAs that originated from pre-mRNAs are thought to be the main actors in the regulation of gene expression [55,56]. However, current evidence indicates that intronic lncRNAs that exhibit independent transcription might also be biologically significant [24,57-59]. Although the ssRNA-seq technique used in this study cannot determine whether these intronic lncRNAs are alternative splicing products of a pre-mRNA or independent transcripts, METH-regulated sense intronic lncRNAs were found to originate from the intronic regions of the corresponding protein-coding genes, such as calcium/calmodulin-dependent protein kinase IV (*Camk4*), cAMP response element-binding protein 1(*Creb1*), CREB-binding protein (*Crebbp*), glutamate receptor, ionotropic, AMPA1 (alpha 1) (*Gria1*) and mitogen-activated protein kinase 10(*Mapk10*), which have been suggested to be responsible for synaptic transmission and specific signal transduction in long-term drug-induced neuroadaptation. Further experiments are needed to investigate the precise natures of these sense intronic lncRNAs. These results suggest that the sense intronic lncRNAs might play an important role in the regulation of METH-induced locomotor sensitization.

## Conclusions

In summary, we reported a transcriptional profiling of lncRNAs in the NAcs of METH-sensitized and control mice. We have identified a number of METH-responsive lncRNAs. The predicted *cis*- and *trans*-associated genes of these METH-regulated lncRNAs were significantly enriched during the cellular and molecular events that contribute to reward and addiction. Although further experiments are needed to investigate the distinct function and the precise regulatory mechanism of each candidate lncRNA, our data suggest that exposure to METH elicits a global alterations in lncRNA expression in the NAc of sensitized mice that might be involved in METH-induced locomotor sensitization and addiction.

## Methods

### Animals

Adult wild-type C57BL/6 mice (7–8 weeks old, male, 20-25 g), purchased from Beijing Vital River Laboratory Animal Technolxogy Co. Ltd were used for these experiments. The mice were kept maintained in a regulated environment (23 ± 1°C, 50 ± 5% humidity) on a 12-h light/dark cycle (lights on from 7:00 am to 7:00 pm) and were handled in accordance with the Institutional Animal Care and Use Committee of Xi’an Jiaotong University. All efforts were made to minimize the number of animals used and to reduce stress from handling during the injections.

### Drugs

The METH hydrochloride used for the tests was purchased from the National Institute for the Control of Pharmaceutical and Biological Products (Beijing, P.R. China), and was dissolved in 0.9% physiological saline. The volume of the intraperitoneal (i.p.) injections was 10 ml/kg.

### Procedure of METH exposure

The treatment regimens used in the current study have been shown to produce robust locomotor sensitization in our previous studies [2,6]. Briefly, the mice were given once-daily injections of saline for two consecutive days (day 1–2), after which they were randomly divided into two groups. The groups of mice were then given once-daily injections of METH (2 mg/kg) or saline for five consecutive days (day 3–7) followed by two injection-free days (day 8–9). On day 10, the mice were given a challenge injection of either 2 mg/kg METH or saline. Horizontal locomotor activity was performed on all drug treatment days for 60 minutes before and after the injections. The injections were performed in the open field test apparatus and during the light phase of the light/dark cycle. On the drug injection days, the mice were brought into the behaviour room 60 min prior to the beginning of the experiments to acclimate to the new environment. For all experiments, the mice were sacrificed 24 h after the final injection.

### NAc sample preparation and RNA isolation

After the mice were sacrificed, the brains were micro-dissected and the NAcs were harvested and immediately frozen in liquid nitrogen. NAc lysates from eight mice from each group were pooled for total RNA isolation, following the instructions of the manufacturer of TRIzol (Invitrogen, USA). One saline and one METH sample were prepared from the RNAs that were extracted from the pooled NAc lysates and these samples were referred to as the saline and METH samples. The RNA qualities were evaluated with an Agilent 2100 BioAnalyzer (Agilent Technologies, USA), and all samples exhibited an RIN > 8.

### Strand-specific cDNA library construction and sequencing

After the total RNA passed the RNA quality control for deep sequencing, we prepared to construct the strand-specific cDNA libraries. Briefly, the total RNA (5 μg) from each sample was fragmented into ~200 base pair (bp) units using a Covaris-S2 system after the removal of the rRNA without preselecting the mRNA. Next, the RNA fragments were used to generate double-stranded cDNA. The first cDNA strand was synthesized using random hexamers, and the second strand of cDNA was synthesized using deoxy-UTP instead of deoxy-TTP with DNA polymerase I. Then the double-stranded cDNAs were end-repaired after purification with a QiaQuick PCR kit, and the adapters were ligated. Subsequently, the uridine-containing strand was destroyed by uracil-N-glycosylase, which enabled the identification of the transcript orientation. Subsequently, to acquire the sequencing library products, the single-stranded adapted cDNA fragments of 200 bp were recovered and purified with agarose gel electrophoresis and then enriched by PCR for 12 cycles. The purified cDNA library products were evaluated using the Agilent 2100 BioAnalyzer and then sequenced on an Illumina HiSeq 2000.

### SsRNA-seq data analyses

After sequencing, the raw reads that were generated by sequencers, were saved in the fastq format. To obtain reliable clean reads, the dirty raw reads were filtered according to four criteria: reads with sequence adaptors were removed; reads with more than 5% ‘N’ bases were removed; low-quality reads, in which more than 50% of the QA were ≤ 15 bases were removed; and ribosomal RNA sequences that were obtained from the ribosomal RNA database SILVA [60] by the software SOAP v2.2.0 [61] were removed based on an allowance of no more than three mismatched bases. All subsequent analyses were based on clean reads. The clean reads of the saline and METH groups were separately aligned to the mouse genome, UCSC mm9 [20] using the software TopHat v2.0.4 [62]. Mismatches of no more than 5 bp were allowed in the alignment of each read. The resulting alignment data from TopHat were then assembled into transcripts by the assembler Cufflinks v2.0.0 [63]. The assembled transcripts that corresponded to known lncRNAs were determined by perfect sequence matching to the NONCODE v3.0 database of known non-coding RNA [21]. Furthermore, the assembled transcripts were aligned to protein databases, including KEGG Orthology [64], non-redundant protein database [65], COG [66] and UniProtKB/Swiss-Prot [67], to obtain protein-coding transcripts. To obtain novel non-coding transcript candidates, the remaining transcripts that were not matched to any known lncRNAs or protein-coding sequences were then used to predict their abilities to encode proteins using the Coding Potential Calculator (CPC) [68], which based on framefinder. Thus, the transcripts that lacked the ability to encode proteins were considered as novel lncRNA candidates.

The clean reads that were uniquely mapped to lncRNAs were used to calculate the expression levels. The relative expression levels of the lncRNAs in the saline and METH groups were measured as the number of uniquely mapped reads per kilobase per million mappable reads (RPKM). The formula was defined as follows: RPKM = 10^6^ × C/(NL × 10^−3^), where C was the number of reads that uniquely mapped to the given transcript, N was the number of reads that uniquely mapped to all transcripts, and L was the total length of the given transcript. The RPKM method eliminates the influences of different transcript lengths and sequencing discrepancies on the calculation of expression. Therefore, the RPKM value was directly used to compare the differences in lncRNA expressions between samples. The fold change from the normalized expression was calculated as log_2_ (RPKM_METH_/RPKM_saline_) to assess the levels. Because we only had one replicate per group, the variances of regulated levels were directly estimated from the RPKM values using the Poisson distribution, and *P*-values were calculated [69]. Therefore, to compensate for false-positive findings at each significance threshold, we calculated a false discovery rate (FDR; Benjamini-Hochberg) for each lncRNA and applied it for genome-wide corrections [70]. We identified lncRNAs that were differentially regulated between the METH and saline groups based on the following criteria: *P* < 0.001, FDR ≤ 0.0001 and absolute value of the fold change ≥ 1.25.

### *Cis* and *trans* analyses

Identification of the genes associated with differentially expressed lncRNAs via *cis*-or *trans*-regulation might provide insight into the potential functions of lncRNAs. We subjected the significantly changed known lncRNAs to *cis* and *trans* analyses. For the *cis* analyses, we classified the differentially expressed lncRNAs into the following 5 categories according to their genomic contexts relative to protein-coding genes: sense intronic, overlapping, NAT, lincRNA and bidirectional. For the *trans* analyses, we predicted the *trans*-associated genes of the differentially expressed lncRNAs with RNAplex v0.2, which is a fast tool for RNA-RNA interaction searches by neglecting intramolecular interactions and by using as lightly simplified energy model [39]. The RNAplex parameters were set as –e < −20 in the current study to identify the *trans*-associated genes [9], and genes that were found to be located on that same chromosome as the lncRNA were excluded.

### GO and KEGG pathway enrichment analyses

For the GO and KEGG pathway enrichment analyses, the *cis*- and *trans*-associated genes of the lncRNAs that were significantly modified in the METH-sensitized mice were analysed with the functional annotation tool Blast2GO [71]. First, all associated genes were mapped to GO terms and pathways in the gene ontology [72] and KEGG pathway [73] databases by calculating the gene numbers for every term and pathway. Then the *P* values were calculated via hypergeometric tests and go through a correction with FDR. *P* < 0.05 and FDR < 0.05 were used as thresholds for defining significantly enriched GO terms and pathways. Finally, the associated genes that corresponded to specific biological functions were filtered.

### QPCR analyses

QPCR was performed on the RNAs isolated from the NAcs of individual mice (n = 11-15 per group). First-strand cDNA was synthesized using the Thermo Scientific RevertAid First Strand cDNA Synthesis Kit (Thermo Scientific, USA). Based on the manufacturer’s instructions and suggested parameters (25°C 5 min, 42°C 60 min, and 70°C 5 min), 500 ng of total RNA from each sample was utilised. QPCR was performed on Bio-Rad iQ™5 system real-time detection instrument (Bio-Rad, USA) using SYBR Premix Ex Taq II (TaKaRa Biotechnology, Japan) in the following conditions: 95°C for 30 sec; 95°C for 10 sec and 60°C for 1 min, which were repeated for 40 cycles. GAPDH was used as an endogenous control for the qPCR, and the relative expression levels were determined by the 2^-△△Ct^ method. Fold changes in expression were calculated with a log 2 transform. Independent-sample *t*-tests were used to test for significant differences (SPSS v17.0, SPSS Inc., USA). The primer pairs are shown in Table 3. Pearson’s coefficient analysis was also performed with SPSS (version 17.0, USA).

**Table 3:** **List of qPCR primers used to validate the sequencing data**

**LncRNA**		**Primer sequence**	**Tm(°C)**	**Product length (bp)**
AK036791	F	5′-TGGTGATTCTGTTACCGTCT-3′	60	134
	R	5′-AGGAGGTCCAAATACAAGAT-3′	58	
AK041753	F	5′-CTGTCCCTTGGTGATGCTGTT -3′	62	132
	R	5′-TGCACTGAAGCATTCTCTCTCC -3′	62	
AK046893	F	5′-CTCCTCCTCCTGGGAATGTCT -3′	62	89
	R	5′-CCCTAAATGTTCTCCTCCGTCTT -3′	62	
AK053003	F	5′-CTCAGGAGGTCATTCGG -3′	50	199
	R	5′-GAGAAACCTGGAGTAGTGG -3′	49	
AK034848	F	5′-TGATAAGGAGGGCGTCACAA -3′	62	149
	R	5′-ACCTTCACCAGCACCCAGA -3′	62	
AK019917	F	5′-CCCCTCTAAGCCTGGGAACT-3′	62	133
	R	5′-AACAAACCAACAAAAGCCACCT-3′	62	
AK085108	F	5′-AAACCAACAACAGGACCTCT-3′	61	179
	R	5′-ATTGGGAGTTTGATGCTTTC-3′	62	
AK048817	F	5′-AGGATGTGAGTGGACTGTGG-3′	62	148
	R	5′-TTTGGTTGTCAGAGATGGCT-3′	62	
AK081238	F	5′-TGTTGTGGGTATATGTGGAT-3′	58	137
	R	5′-ACAGTGAAATAAGATGGACC-3′	56	
AK080587	F	5′-AGCAGAGGATGTATCAAAGC-3′	59	109
	R	5′-ACCAAAGGGACTGACAGAAT-3′	60	
AK003846	F	5′-GTGTGCTCCAGAAAGTGTAA-3′	58	131
	R	5′-GAGAAGAAGAGAGAGGTTGC-3′	58	
AK142426	F	5′-TTTTGGGAGGGTGAGGG -3′	56	177
	R	5′-GAACGGTGAAGGCGACA-3′	55	
GAPDH	F	5′-TGTGTCCGTCGTGGATCTGA-3′	55	150
	R	5′-TTGCTGTTGAAGTCGCAGGAG-3′	52	

The primer pairs were chosen to maintain the melt temperature (Tm) between 45°C and 62°C and the product lengths between 80 and 200 bp.

### Availability of supporting data

The raw sequences have been deposited in the ArrayExpress database (www.ebi.ac.uk/arrayexpress) under accession number E-MTAB-2843.

## Additional files

Additional file 1:
**Full list of the METH-regulated lncRNAs.**
Additional file 2:
**Full list of the sense intronic lncRNAs that were significantly regulated by METH.**
Additional file 3:
**Full list of the overlapping lncRNAs that were significantly regulated by METH.**
Additional file 4:
**Full list of the NATs that were significantly regulated by METH.**
Additional file 5:
**Full list of the lincRNAs that were significantly regulated by METH.**
Additional file 6:
**Full list of the bidirectional lncRNAs that were significantly regulated by METH.**
Additional file 7:
**Full list of the one-to-one** ***trans*** **-acting lncRNAs that were significantly regulated by METH.**
Additional file 8:
**Full list of the GO and pathways of the** ***cis-*** **acting lncRNAs and their associated genes.**
Additional file 9:
**Full list of the GO and pathways of the** ***trans*** **-acting lncRNAs and their associated genes.**

## Abbreviations

Camk4: Calcium/calmodulin-dependent protein kinase IV
CPC: Coding potential calculator
Creb1: cAMP response element-binding protein 1
Crebbp: CREB-binding protein
FDR: False discovery rate
GO: Gene ontology
Gria1: Glutamate receptor, ionotropic, AMPA1 (alpha 1)
Kcnq1: Potassium voltage-gated channel, subfamily Q, member 1
Kcnq1ot1: Potassium voltage-gated channel, subfamily Q, member 1, opposite strand/antisense transcript 1
lincRNA: Long intergenic non-coding RNA
lncRNA: Long non-coding RNA
LTD: Long-term depression
LTP: Long-term potentiation
Mapk10: Mitogen-activated protein kinase 10
METH: Methamphetamine
Miat: Myocardial infarction associated transcript
NAc: Nucleus accumbens
NAT: Natural antisense transcripts
ncRNA: non-coding RNA
Neat1: Nuclear enriched abundant transcript 1
Neat2: Nuclear-enriched abundant transcript 2
qPCR: quantitative real-time PCR
RPKM: Reads per kilobase per million mappable reads
ssRNA-seq: strand-specific complementary DNA sequencing technology
Zfhx2: Zinc finger homeobox 2
Zfhx2as: Zinc finger homeobox 2, antisense
